# Physico-chemical characterization of nano-emulsions in cosmetic matrix enriched on omega-3

**DOI:** 10.1186/1477-3155-9-41

**Published:** 2011-09-21

**Authors:** Tin-hinan Kabri, Elmira Arab-Tehrany, Nabila Belhaj, Michel Linder

**Affiliations:** 1 Laboratoire d'Ingénierie des Biomolécules (LIBio), Nancy-Université, 2 avenue de la Forêt de Haye, 54500 Vandoeuvre-lès-Nancy, France

## Abstract

**Background:**

Nano-emulsions, as non-equilibrium systems, present characteristics and properties which depend not only on composition but also on their method of preparation. To obtain better penetration, nanocosmeceuticals use nano-sized systems for the delivery of active ingredients to targeted cells. In this work, nano-emulsions composed of miglyol, rapeseed oil and salmon oil were developed as a cosmetic matrix. Measurements of different physico-chemical properties of nano-emulsions were taken according to size, electrophoretic mobility, conductivity, viscosity, turbidity, cristallization and melting point. The RHLB was calculated for each formulation in order to achieve maximum stability.

**Results:**

Both tween 80 and soya lecithin were found to stabilize formulations. The results showed that rapeseed oil and miglyol are the predominant parameters for determining the expression of results concerning the characterization of emulsion. Based on the mixture design, we achieved the optimal point using the following formulation: 56.5% rapessed oil, 35.5% miglyol, and 8% salmon oil. We considered this formulation to be the best as a nanocosmeceutical product due to the small size, good turbidity, and average HLB.

**Conclusions:**

This study demonstrates the influence of formulation on the physico-chemical properties of each nano-emulsion obtained by the mixture design.

## Introduction

Cosmetics represent an important industry worldwide, especially in Europe. The matrix of cosmetics is not simple; it usually contains a high number of ingredients and often requires time-consuming and tedious sample treatments [1,2].

Nanotechnology is a key technology leading to product innovation. Nano-emulsions have attracted considerable attention in recent years as potential vehicles for the controlled delivery of cosmetics and personal care products [3].

Nano-emulsions are oil-in-water (O/W) or water-in-oil (W/O), transparent or translucent, colloidal dispersions, usually in the 20-500 nm size range [4,5]. An advantage in using nano-emulsions compared to ordinary emulsion, is their ability, as delivery systems, to improve the bioavailability and bioefficacy of lipophilic bioactives in their delivery [6]. The very small droplet size causes a large reduction in the force of gravity, and the Brownian motion may be sufficient for overcoming it. This means that no creaming, sedimentation and flocculation occurs during storage. Weak flocculation is prevented, which enables delivery system to remain dispersed with no separation [7].

Nano-emulsions render possible different visual aspects, richness and skin feel in a great variety of products such as lotions, transparent milks and crystal-clear gels with different rheological behavior, properties which themselves represent biophysical and sensorial benefits highly valued by consumers [8].

Eicosapentaenoic acid (EPA, 20:5*n*-3) and docosahexaenoic acid (DHA, 22:6*n*-3) are two major polyunsaturated fatty acids (PUFA), not present in normal skin epidermis. However, they are metabolized by skin epidermal enzymes into anti-inflammatory and anti-proliferative metabolites that are associated with a variety of benefits regarding inflammatory skin disorders [9]. Oil and marine lecithin from salmon (*Salmo salar*) contains a high percentage of PUFAs, especially EPA and DHA [10,11].

This study is focused on the development of a cosmetic matrix made of nano-emulsions enriched on long-chain polyunsaturated fatty acids (LC-PUFA) as final application products, and on the optimization of their preparation. We focused on two of them: eicosapentaenoic acid (EPA, 20:5*n*-3) and docosahexaenoic acid (DHA, 22:6*n*-3).

We formulated a mixture of various oils (rapeseed, salmon and miglyol) based on an innovative design. This formulation helps us mask the salmon oil smell. We calculated the required hydrophilic-lipophilic balance (RHLB) for each experiment and measured different physico-chemical parameters of each nano-emulsion, such as size, electrophoretic mobility, viscosity, turbidity, crystallization and melting point.

## Materials and methods

The salmon oil (*Salmo salar*) was provided by enzymatic hydrolysis. The lipids were extracted through a low temperature enzymatic process without using any organic solvent [12]. BF_3 _(Boron trifluoride)/methanol (purity = 99%) and hexane (purity = 97%) used for gas chromatograph (CG) with a flame ionization detector were purchased from Fisher (France). These organic solvents were analytical grade reagents. We used Rapeseed oil (Walter Micher, Ferme d'Ormes, France), Caprylic/Capric Triglyceride (Miglyol 812, SASOL, Paris, France), Soya lecithin (Lecrsoyaf F60 IP, France) and Polyoxyethylene Sorbitan Monooleate (Tween 80, Sigma-Aldrich, Munich, Germany) to prepare the nano-emulsions.

### Mixture design

To assess the effects and interactions between different constituents of Scheffe design, we constructed a mixture design with 13 experiments, each containing a different percentage of rapeseed, salmon and miglyol oils, and representing different cosmetic matrices (candidate points). All 13 emulsions were prepared in our laboratory. We used the NEMROD^® ^software (New Efficient Methodology for Research using Optimal Design) to generate the mixture design [13].

The proportions of the various components in the emulsions are (wt):

• 30% of oily phase

• 12% of emulsifiants

• 3% glycerol

• 55% aqueous phase (water)

We developed a mixture design with constraint [14], Table 1, to predict the dependence of observed responses on the cosmetic matrix compositions.

**Table 1:** Lower and Upper Constraint for each ingredient in oil phase

Oil	Lower Constraint (%)	Upper Constraint (%)
Rapeseed oil	20	80

Miglyol	20	80

Salmon oil	2	10

The proportions of emulsifiers (tween 80 and soya lecithin) vary in relation to the proportions of oils (miglyol, rapeseed and salmon) used for the mixture design.

Data from the physico-chemical properties were used to calculate the special cubic equation for the three components:

(1) $$\text{Y}={\text{b}}_{1}{\text{X}}_{1}+{\text{b}}_{2}{\text{X}}_{2}+{\text{b}}_{3}{\text{X}}_{3}+{\text{b}}_{12}{\text{X}}_{1}{\text{X}}_{2}+{\text{b}}_{13}{\text{X}}_{1}{\text{X}}_{3}+{\text{b}}_{23}{\text{X}}_{2}{\text{X}}_{3}+{\text{b}}_{123}{\text{X}}_{1}{\text{X}}_{2}{\text{X}}_{3}$$

in which Y is the measured response, b(i) are parameters estimated for each linear and cross-product term in the model, and X1, X2 and X3 represent level constituents of rapeseed oil, miglyol and salmon oil.

Table 2 presents the mixture design used for the cosmetic matrix.

**Table 2:** Mixture plan used for the cosmetic matrix

N° Experience	Rapeseed oil	Miglyol	Salmon oil
1	0.7800	0.2000	0.0200

2	0.7000	0.2000	0.1000

3	0.2000	0.7800	0.0200

4	0.2000	0.7000	0.1000

5	0.7400	0.2000	0.0600

6	0.4900	0.4900	0.0200

7	0.4500	0.4500	0.1000

8	0.2000	0.7400	0.0600

9	0.4700	0.4700	0.0600

10	0.6250	0.3350	0.0400

11	0.585	0.3350	0.0800

12	0.3350	0.6250	0.0400

13	0.3350	0.5850	0.0800

### Determination of required hydrophilic-lipophilic balance (RHLB) in the oil mixture

Hydrophilic-lipophilic balance (HLB) was calculated using the Griffin empirical method translating into the following formula [15]:

(2) $$HLB=20(1-\frac{Is}{Ia})$$

where **Is is the **saponification Index, and **Ia, the **acid index, both of which were measured based on oil indexes analysis.

We calculated the RHLB using the following equation:

(3) $$RHLB=\left(x1∕X\right).HLB_{rapeseed}+\left(x2∕X\right).HLB_{miglyol}+\left(x3∕X\right).HLB_{salmon}$$

in which x_1, 2, 3 _is the proportion of rapeseed oil, salmon oil and miglyol, and X, the total proportion of oil phase in the formulation.

### Oil indexes analysis

We used standard procedures of the American Oil Chemists' Society to arrive at the index values [16]: acid index (standard 969.17 1997) and saponification index (standard 920.160, 1997).

### Fatty acid composition

Fatty acid methyl esters (FAMEs) were prepared [17]. To separate the FAMEs, we used a Perichrom™ 2000 gas chromatograph (Perichrom, Saulx-lès-Chartreux, France), equipped with a flame-ionization detector, and a fused silica capillary column (50 m, 0.25 mm i.d. **× **0.25 μm film thicknesses, CP 7419 Varian, Middelburg, Netherlands). Injector and detector temperatures were set at 260°C. A temperature program of column was initially set at 145°C for 5 min, to rise to 210°C at a rate of 2°C/min and to be held at 210°C for 10 min. We used standard mixtures (PUFA1 from a marine source and PUFA2 from a vegetable source (Supelco, Sigma-Aldrich, Bellfonte, PA, USA) to identify fatty acids. Results were presented as triplicate analyses.

### Preparation of nano-emulsions

We initially mixed the three oils (miglyol, rapeseed and salmon) in different proportions based on the mixture design. The appropriate quantity of soya lecithin was determined based on the RHLB added in the oil phase and mixed during 5 min by vortex in inert atmosphere (nitrogen).

We mixed the glycerol (3%) and tween 80 with water (55%) at 50°C and then under vortex.

The oil phase added into aqueous phase and then we sonicated the mixture at 40 kHz and 40% of full power, for 120 s (1 s on and 1 s off), to achieve a homogeneous solution.

To obtain the various nano-emulsions based on the mixture design, we circulated the O/W emulsions five times through a high-pressure valve homogenizer Emulsiflex-C3 (provided by Sodexim S.A, France), at 22,000 psi. The homogenization temperature was kept at 10°C by circulating cold water (4°C) around the valve. Each re-circulated sample was kept under nitrogen atmosphere to avoid any oxidation. Emulsion samples were stored in the dark in sterilized bottles at 20°C.

### Size measurement of droplet emulsions

Oil droplet sizes were analyzed by dynamic light scattering using a Malvern Zetasizer Nano ZS (Malvern Instruments Ltd, UK). The apparatus is equipped with a 4 mW He/Ne laser, emitting 633 nm, measurement cell, photomultiplier and correlator. Prior to measuring size, the samples were diluted (1:400) into a distilled water ultra-filtrate and placed in vertical cylindrical cuvettes (10 mm-diameter). Using an avalanche of photodiode detector set at 25°C, we measured the scattering intensity at a 173° angle relative to the source. Intensity autocorrelation functions were analyzed by a General Purpose Algorithm (integrated into the Malvern Zetasizer software) in order to determine the distribution of the translational z-averaged diffusion coefficient of the particles, D_T _(m^2 ^s^-1^). The D_T _parameter and the hydrodynamic radius (R_h_) of particles are related through the Stokes-Einstein equation: D_T _= k_B_T/6πηR_h_. In dispersion, particles move in a constant random Brownian motion such as it causes the intensity of scattered light to fluctuate as a function of time. Therefore droplets' sizes were measured based on the correlation function as established by the dispersion technology software (DTS), using various algorithms. The refractive index (RI) and absorbance were set respectively at 1.471 and 0.01 at 25°C. Measurements were repeated five times.

### Electrophoretic Mobility

Electrophoretic mobility measurements (μE) were performed by means of laser Doppler electrophoresis. Each sample was put in a standard capillary electrophoresis cell equipped with gold electrodes. The emulsions were diluted (1:400) to measure their electrophoretic mobility using a Malvern Zetasizer Nano ZS (Malvern instruments, UK) to evaluate the surface net charge around the lipid droplets. Prior to analysis, emulsions were diluted with de-ionized water, to avoid multiple scattering effects, and were then directly placed into the equipment. Measurements were performed directly in the diluted emulsions (mean, n = 3).

### Turbidity

The experiments were carried out in a 50-ml bottle. Temperature was maintained at 20°C and a turbidity sensor positioned through the bottle wall, to avoid disturbances during stirring. Turbidity changes accompanying the nano-emulsion formulation were observed using a turbidity meter (Analite NEP 160, McVan Instruments, Mulgrave, Australia). This apparatus used light in the near infrared region (860 nm) so that any particle in suspension in the fluid reflected the incident beam at 180° back to a sensitive electronic receptor. A measurement system ensured the continuous monitoring of turbidity (Almemo 8990-8 V5, Ahlborn, Holskirchen, Germany). This data logger was coupled with a PC equipped with an AMR WinControl software (adapted for Almemo). Data were collected automatically every second during 1,000 seconds, then every five seconds. All runs were carried out at least in triplicate.

### Measuring system for the rheological behavior

The Stress Tech Rheometer (Reologica AB, Lund, Sweden) was equipped with a C40.4 cup.

All runs were carried out at least in duplicate. The applied constraint ran from 0.2 to 1 Pa.s on 25 points. Integration and acquisition time was of 10 seconds with automatic resolution on each measurement cycle, itself performed in triplicate.

### Differential scanning calorimetry

Differential Scanning Calorimetry (DSC) technique was applied with a DSC 204F1 NETZSCH (France). First, an emulsion sample of about 10 mg was placed in an aluminum pan, hermetically sealed before being placed in the calorimeter thermocouples.

The samples were then heated from 20°C to 60°C in 5°C/min heating increments, to eliminate initial thermal history (equilibrated at 60°C), and then cooled down to -60°C following the same process (equilibrated at -60°C). The samples were then reheated to 60°C, in 5°C/min heating increments as well.

### Color measurement

The color of the oils was determined in triplicate, using the Lovibond method. Color was measured using the Lovibond (Lovibond PFX195, VWR International France). The equipment was calibrated following the method described by Windsor and Barlow [18], fixing the color yellow in 30 units and varying the color red. Each sample was taken in a cube and placed in the space provided in the tintometer. We proceeded by trial and error to match different combinations of red and yellow slides with the reflected color of the oil, until we did.

## Results and Discussion

### Fatty acid analyses

The samples' main fatty acid composition is shown in Table 3. Salmon oil and rapeseed oil exhibited the highest percentage of total polyunsaturated fatty acids. In regards to salmon oil, the most significant proportions of fatty acids were respectively of C22:6 and C20:5, found in the polyunsaturated fatty acids class, C18:1(n-9), found in the monounsaturated fatty acids class, and C16:0, found in the saturated fatty acids class. In regards to rapeseed oil, the percentage of C18:3n-3 was important in the polyunsaturated fatty acids class. The fatty acid most present was C18:1n-9 with 55.47%, found in the monounsaturated fatty acids class. Table 3 shows miglyol as containing a high percentage of saturated fatty acids such as C8:0 and C10:0.

**Table 3:** Main fatty acid compositions of different oils by gas chromatography (area %)

Fatty acids	Rapeseed oil		Miglyol		Salmon oil	
	**%**	**sd**	**%**	**sd**	**%**	**sd**

**C6:0**	0.00	0.00	0.16	0.03	0.00	0.00

**C8:0**	0.00	0.00	55.09	0.81	0.00	0.00

**C10:0**	0.00	0.00	41.47	0.14	0.00	0.00

**C12:0**	0.00	0.00	0.21	0.01	0.00	0.00

**C14:0**	0.00	0.00	1.87	0.06	2.40	0.02

**C16:0**	4.39	0.35	0.00	0.00	15.34	0.18

**C18:0**	1.70	0.55	0.00	0.00	4.35	0.07

**C20:0**	0.53	0.03	0.00	0.00	0.00	0.00

**SAT**	**6.62**	**-**	**98.75**	**-**	**22.09**	**-**

**C16:1 n-9**	0.16	0.00	0.00	0.00	2.55	0.03

**C18:1 n-9**	59.76	0.67	0.00	0.00	23.18	0.33

**C18:1 n-7**	3.18	0.20	0.00	0.00	2.66	0.08

**C20:1**	1.21	0.12	0.00	0.00	-	-

**MONO**	**64.31**	**-**	**0.00**	**-**	**28.39**	**-**

**C18:2 n-6**	19.27	0.25	0.00	0.00	6.77	0.01

**C20:3 n-6**	0.00	0.00	0.00	0.00	0.00	0.00

**C20:4 n-6**	0.00	0.00	0.00	0.00	1.61	0.03

**C22:4n-6**	0.00	0.00	0.00	0.00	0.19	0.01

**n-6**	**19.27**	**-**	**0.00**	**-**	**8.57**	**-**

**C18:3 n-3**	9.53	0.07	0.00	0.00	2.64	0.03

**C18:4 n-3**	0.00	0.00	0.00	0.00	-	-

**C20:4 n-3**	0.00	0.00	0.00	0.00	-	-

**C20:5 n-3**	0.00	0.00	0.00	0.00	7.35	0.01

**C22:5 n-3**	0.00	0.00	0.00	0.00	-	-

**C22:6 n-3**	0.00	0.00	0.00	0.00	17.48	0.09

**n-3**	**9.53**	**-**	**0.00**	**0.00**	**27.47**	**-**

**PUFA**	**28.80**	**-**	**0.00**	**0.00**	**36.04**	**-**

**n-6/n-3**	**2.02**	**-**	**0.00**	**0.00**	**0.31**	**-**

Data obtained are the average ± standard deviation in triplicate.

sd: standard deviation

### Results of emulsions characterization

We used the NEMROD^® ^software with a cubic model to analyze experimental results. At this stage of the experiment, we focused on size distribution, electrophoretic mobility, HLB and turbidity values in emulsions' system. We investigated the impact of formulation on physico-chemical properties of emulsions. Table 4 presents the coefficients of our model for each response.

**Table 4:** Estimation of model coefficients (Equation 1)

Coefficient	Size distribution of emulsion (nm)	Mobility electrophoretic (μmcm/Vs)	HLB	Turbidity (NTU)*	Melting onset (°C)
b1	192.520*	-5.053**	7.543***	48761.993***	-23.31

b2	276.223*	-5.605**	12.674***	18764.217*	20.87

b3	6582.411	-176.643	-91.207	528043.223	7849.77***

b12	-327.298	6.421	5.867	-91049.791*	14.03

b13	-7249.278	212.419	127.855	-884315.817	-8481.72**

b23	-8417.213	218.825	143.770*	-709495.692	-8998.93**

b123	2206.082	-105.357	-59.960	898519.743	-233.36

R^2^	0.89	0.82	0.99	0.95	0.95

Meaning of each star: * 95%, ** 99%, *** 99.9% confidence level

*NTU: Nephelometric Turbidity Units (NTU)

b1: rapeseed oil; b2: miglyol; b3: salmon oil

**Figure 1:**
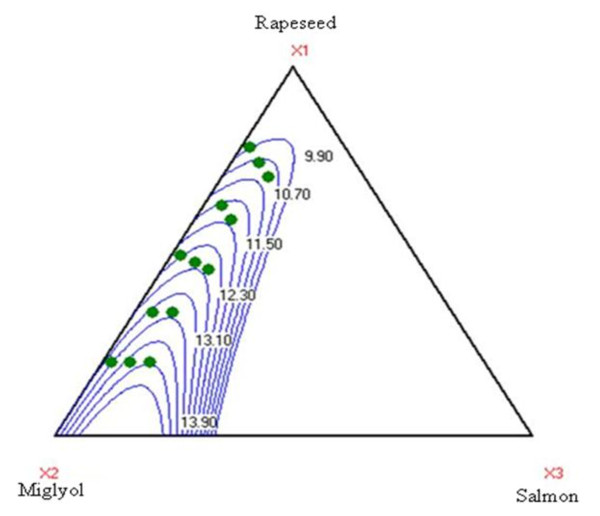
**Variation of HLB in function of different oil mixture**.

Table 4 shows expression of results concerning the characterization of emulsion (size distribution, mobility electrophoretic, HLB, turbidity and melting point): rapeseed oil and miglyol are the determining parameters. Rapeseed oil has a significant influence on HLB and on turbidity parameters, with a 99.9% confidence level, and with a lesser one on electrophoretic mobility, size distribution and melting onset. Figure 1, which presents results of the degree of influence of miglyol and rapeseed oil on HLB response, shows that miglyol plays a major role on HLB of emulsions.

Figure 1. Variation of HLB based on different oil mixtures.

Results show that the influence of salmon oil is very important on the melting onset. In addition, interactions between rapeseed oil/salmon oil and miglyol/salmon oil are noticeable.

We can increase emulsion stability by decreasing droplet size. In this study, the optimal size distribution is 130 ± 10 nm for an HLB of 12.5 ± 0.08.

The distribution of droplet size is related to the polydispersity index (PdI). When this index is lower than 0.25, all droplets have a similar size [19]. Study results show an average PdI of 0.16 ± 0.046, indicating a good stability of the emulsions.

For a nano-emulsion at 130 nm, the turbidity factor is about 9900 NUT (Nephelometric Turbidity Units) and appears correlated to the proportion of rapeseed oil present in the emulsion. In addition, we established we could decrease turbidity to 8000 NTU by increasing the proportion of miglyol in it.

Using our mixture design, we arrived at the optimal point based on the following formulation:

56.5% of rapeseed oil, 35.5% of miglyol, and 8% salmon oil. Table 5 shows the calculated and experimental values of different characteristics of this emulsion: there is no significant difference between calculated and experimental values except in regards to turbidity.

**Table 5:** Experimental and calculated values of the different response

Response	Model value	Experimental value
Size (nm)	136.38	139.74 ± 1.09

Mobility electrophoretic (μmcm/Vs)	-3.56	-4.90 ± 0.28

Turbidity (NTU)	12493	16373 ± 200

HLB	11.54	10.76 ± 1.02

Melting onset (°C)	-15.9	-16.5 ± 0.93

### Droplet size analysis

We measured the particle sizes of different nano-emulsions stabilized by soya lecithin and tween 80, immediately after homogenizing. The minimum size that can be achieved generally depends on the viscosity of materials used and on applied homogenization parameters (number of cycles and pressure). In our study, the average droplet size was 143 nm and the polydispersity index was 0.16. To obtain a distribution of centered size, we had to perform five successive cycles of homogenization, hence demonstrating that the size of the nano-droplets depends not only upon such physical parameters as number of cycles and pressure, but also on oil composition and the surface-active properties of the soya lecithin used [20,21].

### Electrophoretic Mobility

Measurements of electrophoretic mobility vary between -3 and -4 μmcm/Vs with a relatively high stability of the formulations. It is mainly due to the fact that the lipophilic and hydrophilic characteristics of the two emulsifiers used in this study maintain the equilibrium between the positive and negative charges.

### Turbidity

The turbidity value stabilized at 6248 NTU in the case of emulsion no. 8. Results showed that turbidity depends upon the percentage of miglyol and rapeseed oil in the formulation.

By increasing the amount of miglyol in the formulation, we observed a decrease in turbidity. Miglyol is fluid and transparent. The decrease in turbidity correlates with miglyol's fluidity and color characteristics.

On the other hand, we observe an increase in turbidity in direct proportion with the quantity of rapeseed oil in emulsion formulation. This phenomenon is related to color and fluidity of the rapeseed oil.

**Figure 2:**
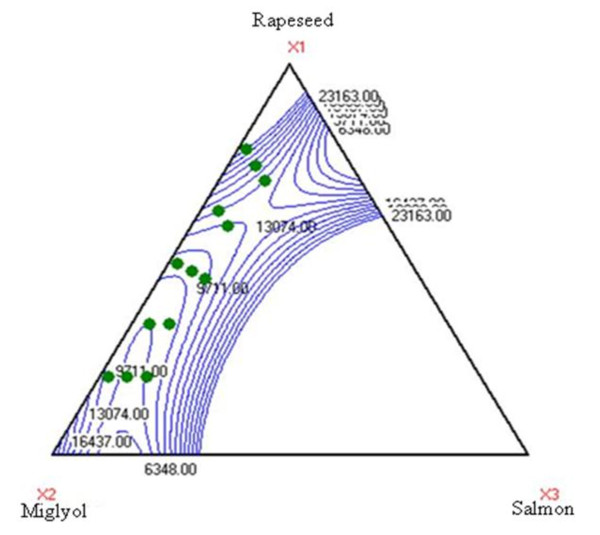
**Variation of turbidity in function of oil type**.

Finally, results showed that salmon oil had no influence on turbidity. Figure 2 shows the significant influence of rapeseed oil and miglyol on turbidity.

Figure 2. Variation of turbidity in function of oil type.

### Rheological properties

Rheological properties of the oils were characterized. Measurements of the shear rate as a function of the shear stress are plotted for rapeseed oil. Curve slope and correlation coefficient are y = 0.017x+0.0003 and 0.99, respectively.

We observed the same behavior for two other matrix (rapeseed oil and miglyol). Rheological properties of oils were determined using a Stress Tech Rheometer: they exhibited a Newtonian flow behavior.

Rheological properties of the 13 emulsions were also characterized. We observed the same behavior for other systems, with a curve slope equal to y = 0.0468x-0.0095 and a correlation coefficient was 0.99. Results lead to the same conclusions and indicate a Newtonian flow behavior.

These results suggest that tween 80 and soya lecithin do not affect the rheological properties of our emulsions.

### Calculation of the RHLB in oil phase

We measured saponification and acid indexes and calculated the HLB of each oil phase using equation 2. Table 6 shows results of saponification and acid indexes and balance lipophilic-hydrophilic (HLB) for rapeseed oil, salmon oil and soya lecithin. The HLB of the Miglyol 812 is 15.36 according to Macedo et al. [22].

**Table 6:** Results of saponification and acid indexes and balance lipophilic-hydrophilic (HLB) for rapeseed oil, salmon oil and soya lecithin

Experimental results	Rapeseed oil	Salmon oil	Soya lecithin
Saponification index	215.98 ± 0.07	201.96 ± 0.16	215.98 ± 0.05

Acid index	151.47 ± 0.56	112.20 ± 0.73	280.50 ± 0.26

Balance Lipophilic -Hydrophilic (HLB)	8.5	16	4.6

Data obtained are the average ± standard deviation in triplicate.

Then RHLB values were calculated using equation 3. For an optimal emulsification, we must adjust the proportions of two emulsifiers, one with a lipophilic tendency (soya lecithin, HLB = 4.6), and the other with a hydrophilic tendency (tween 80, HLB = 15).

### Oil and emulsion characterization by DSC

Due to the dispersion of triacylglycerols (TG) molecules in individual particles [23], crystallization in emulsion droplets and nanoparticles renders the process of nucleation and crystal growth, well-described for bulk phase, even more complex,. In this study, we observed the influence of lipid composition on crystallization and on melting behavior of TG molecules.

**Figure 3:**
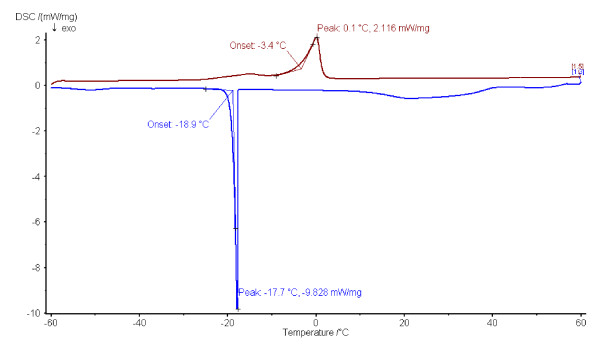
**DSC thermogram showing the crystallization and melting point of a W/O emulsion sample 7 (the red curve presents the melting point and blue curve presents the crystallization point)**.

Thermograms obtained during cooling and heating of bulk oil are presented in Table 7, and the O/W emulsion samples in Figure 3. Analysis results show that, on one hand, the melting points of rapeseed and salmon oils are very close. On the other hand, salmon oil crystallization point sets at -18.7°C and that of rapeseed oil at -54.9°C (Table 7). These data point to the fact that salmon oil changes from liquid to solid state within a very narrow temperature range, near 5°C, while rapeseed oil and miglyol change from liquid to solid at 29°C and 28°C respectively. Considering the ratio between the areas of the peaks in relation with the corresponding amounts of energy released, and therefore with the amount of bulk and dispersed material, it is possible to follow the evolution of the emulsion in time [24].

**Table 7:** Results of melting and crystalization of used bulk oils

Analyzed oil	**Melting temperature (°**C)	**Cristallisation temperature**(°C)
Rapeseed oil	-25.3 ± 0.07	-54.9 ± 0.07

Salmon oil	-24.7 ± 0.82	-18.7 ± 0.42

Miglyol	-2.1 ± 0.35	-30.2 ± 0.28

Data obtained are the average ± standard deviation in triplicate.

Figure 3 shows the evolution of experimental crystallization and melting temperatures determined for emulsion 7.

Figure 3 DSC thermogram shows the crystallization and melting point of a O/W emulsion sample 7.

We can observe that, for each formulation, melting and crystallization temperatures vary in proportion with both oil and emulsifier.

Results show that the beginning of crystallization, represented by the onset on the graph, is close to the total crystallization of the matter. The variation in temperature between primary nucleation and total crystallization of the emulsion is limited: it is of 0.3°C on average. On the other hand, temperature variation is high for all components to reach total melting. It is necessary to use high levels of energy to maintain the emulsion in a state of total melting.

Vanapalli et al. [25] performed a DSC study of the stability of O/W emulsions in food applications. They investigated the effect of oil type, dispersed oil volume fraction and cooling rate on the stability of emulsified. According to Clause et al. [24], the peak area of the thermogram is a revealing index of droplet size distribution: the narrower the area is, the smaller the particle size, and thus, the system polydispersity is lower than 0.25.

### Color measurement

Objective color evaluation performed by reflectance spectrophotometry allows the computation of CIE tristimulus values (X, Y, Z) which can be mathematically transformed into co-ordinates describing color in a more intuitive and easier way [26]. These co-ordinates determine lightness (L*in the CIE L* a* b* or Y in xyY system) and chromaticity (a*, b*in the CIE L* a* b* system; and xy in the xyY system).

Color is an important indication of product composition, purity, and degree of deterioration. It is a quick check on degradation, and on the suitability and stability of a product for a particular use [27]. In this study, we converted the Lab values to xyY using the CIE software, and then identified each oil color on a chromaticity diagram. Results show that Lovibond color is higher in salmon oil (x: 0.45; y: 0.34) than in rapeseed oil (x: 0.40; y: 0.46) and in miglyol (x: 0.33; y: 0.38).

## Conclusion

Nanotechnology is a rapidly expanding and potentially beneficial field with tremendous implications for society, industry, and medicine.

Understanding nano-emulsification processes is of prime importance for the development of nanoparticulate systems. In the formulation of nano-cosmetics, homogenization appears to be the favored method of preparation of the cosmetic matrix in order to reduce the size of emulsion. The use of a mixture design approach appears to be a valuable tool in the investigation of real cosmetic matrix systems consisting of multiple ingredients, the proportions of which, in the mixture, are interrelated. Stable nano-emulsions containing an appropriate amount of emulsifiers were successfully formulated. Different physico-chemical properties of nano-emulsions were measured such as size distribution, mobility, electrophoretic mobility, HLB, turbidity and melting point. The optimal formulation was calculated using a Nemrod software according to these physico-chemical properties. This study indicates the influence of formulation on physico-chemical properties of each nano-emulsion obtained by the mixture design.

## Abbreviation

(EPA): Eicosapentaenoic acid; (DHA): Docosahexaenoic acid; (PUFA): Polyunsaturated fatty acids; (LC-PUFA): Long-chain polyunsaturated fatty acids; (RHLB): Required hydrophilic-lipophilic balance; (CG): Gas chromatograph; (NEMROD): New Efficient Methodology for Research using Optimal Design; (HLB): Hydrophilic-lipophilic balance; (FAMEs): Fatty acid methyl esters; (DTS): Dispersion technology software; (RI): Refractive index; (DSC): Differential Scanning Calorimetry; (PdI): Polydispersity index; (NUT): Nephelometric Turbidity Units; (TG): triacylglycerols.

## Competing interests

The authors declare that they have no competing interests.

## Authors' contributions

THK and NB performed all necessary experiments and analyzed the data collected. EAT and ML validated the mixture design and data. EAT drafted the manuscript. All authors read and approved the final manuscript.
